# The Diminished Expression of Proangiogenic Growth Factors and Their Receptors in Gastric Ulcers of Cirrhotic Patients

**DOI:** 10.1371/journal.pone.0061426

**Published:** 2013-04-19

**Authors:** Jiing-Chyuan Luo, Yen-Ling Peng, Ming-Chih Hou, Kuang-Wei Huang, Hui-Chun Huang, Ying-Wen Wang, Han-Chieh Lin, Fa-Yauh Lee, Ching-Liang Lu

**Affiliations:** 1 Department of Medicine, National Yang-Ming University, School of Medicine, Taipei, Taiwan; 2 Division of Gastroenterology, Taipei Veterans General Hospital, Taipei, Taiwan; 3 Endoscopic Center for Diagnosis and Therapy, Department of Medicine, Taipei Veterans General Hospital, Taipei, Taiwan; 4 Healthcare and Management Center, Taipei Veterans General Hospital, Taipei, Taiwan; University of Navarra School of Medicine and Center for Applied Medical Research (CIMA), Spain

## Abstract

**Objectives:**

The pathogenesis of the higher occurrence of peptic ulcer disease in cirrhotic patients is complex. Platelets can stimulate angiogenesis and promote gastric ulcer healing. We compared the expressions of proangiogenic growth factors and their receptors in the gastric ulcer margin between cirrhotic patients with thrombocytopenia and those of non-cirrhotic patients to elucidate possible mechanisms.

**Methods:**

Eligible cirrhotic patients (n = 55) and non-cirrhotic patients (n = 55) who had gastric ulcers were enrolled. Mucosa from the gastric ulcer margin and non-ulcer areas were sampled and the mRNA expressions of the proangiogenic growth factors (vascular endothelial growth factor [VEGF], platelet derived growth factor [PDGF], basic fibroblast growth factor [bFGF]) and their receptors (VEGFR1, VEGFR2, PDGFRA, PDGFRB, FGFR1, FGFR2) were measured and compared. Platelet count and the expressions of these growth factors and their receptors were correlated with each other.

**Results:**

The two groups were comparable in terms of gender, ulcer size and infection rate of *Helicobacter pylori.* However, the cirrhotic group were younger in age, had a lower platelet count than those in the non-cirrhotic group (p<0.05). The cirrhotic patients had diminished mRNA expressions of PDGFB, VEGFR2, FGFR1, and FGFR2 in gastric ulcer margin when compared with those of the non-cirrhotic patients (p<0.05). Diminished expressions of PDGFB and VEGFR2, FGFR1, and FGFR2 were well correlated with the degree of thrombocytopenia in these cirrhotic patients (ρ>0.5, p<0.001).

**Conclusions:**

Our findings implied that diminished activity of proangiogenic factors and their receptors may contribute to the pathogenesis of gastric ulcers in cirrhotic patients.

## Introduction

Previous studies have shown that cirrhotic patients have a higher prevalence of peptic ulcer disease (PUD) than the general population [1], [2]. Recent large, population-based cohort studies from Taiwan have shown that cirrhotic patients have a higher risk of developing peptic ulcer bleeding (PUB) and ulcer rebleeding [3], [4]. The pathogenesis of the higher incidence of PUD in cirrhotic patients is complex and multi-factorial. Theoretically, portal hypertension is involved which causes splanchnic congestion, impaired reparative processes of the gastro-duodenal mucosa, and gastric micro-vascular abnormalities [5], [6], all of which lead to increased susceptibility to acid and pepsin. It has also been demonstrated that a cirrhotic condition leads to impaired gastric mucosal defense/repairing mechanisms, including impaired bicarbonate and mucus secretion, decreased endogenous prostaglandin synthesis, gastric mucosal blood flow, and diminished mucosal oxygenation [7], [8], [9].

Ulcer formation is a dynamic imbalance between aggressive mucosal factors and defensive/repairing factors. When these defensive and healing factors are less than the aggressive factors, mucosal injuries worsen and ulcers develop [10]. Angiogenesis is a pivotal process in gastric ulcer healing [11]. Several proangiogenic factors are stored in platelets, including vascular endothelial growth factor (VEGF), basic fibroblast growth factor (bFGF), and platelet derived growth factor (PDGF) [12]. These growth factors are released into the ulcer base from platelets and activate and promote the formation of new blood vessels during the hemostasis phase of ulcer healing [13]. This is likely to account for the ability of platelets to stimulate angiogenesis and promote gastric ulcer healing [12], [14]. Thrombocytopenia is usually seen in cirrhotic patients due to splenic platelet sequestration and a reduction in the level and activity of thrombopoietin [15].

In this study, we compared the expressions of proangiogenic growth factors (VEGF, PDGF, bFGF) and their receptors (VEGFR1, VEGFR2, PDGFRA, PDGFRB, FGFR1, FGFR2) over the gastric ulcer margin between cirrhotic patients with thrombocytopenia and those of non-cirrhotic patients without thrombocytopenia. In addition, we investigated the role of proangiogenic factors and their receptors in gastric ulcer healing in cirrhotic patients.

## Materials and Methods

### Patients Enrollment

Cirrhotic and non-cirrhotic patients diagnosed with gastric ulcers proven by video esophagogastroduodenoscopy (EGD) (Olympus GIF-XQ260 gastrointestinal videoscope, Aizo Olympus, Fukushima, Japan) were consecutively enrolled. The diagnosis of liver cirrhosis in this study was based on characteristic findings, including physical stigmata of cirrhosis, computed tomography or ultrasound findings of a nodular liver surface, coarsened echogenicity of liver parenchyma, an enlarged spleen, and the detection of esophageal varices by endoscopy [16]. Exclusion criteria included those taking aspirin, nonsteroidal anti-inflammatory drugs (NSAIDs), clopidogrel, ticlopidine, steroids, proton pump inhibitors, histamine receptor 2 antagonists, misoprostol or sorafenib, and those who drank alcohol or smoked, had thrombocytopenia (platelets <30,000/mm^3^), hepatocellular carcinoma or other malignancy including malignant ulcers, active ulcers with bleeding, a past history of gastric surgery, bleeding tendency, uremia, unstable disease activity, or poor general condition [17]. This study was approved by the Institutional Review Committee of Taipei Veterans General Hospital (TVGHIRB: 98-12-04) and was conducted in accordance with the guidelines of the Declaration of Helsinki. All patients provided written informed consent before participating in this study under the assumption that they had gastric ulcers during EGD.

### Measurements

The patients’ medical and personal histories were reviewed, and laboratory data including platelet count, serum albumin, hemoglobin, and creatinine were recorded. During the EGD procedure, the size of the gastric ulcer was measured using calibrated biopsy forceps [18], and mucosal tissue from the ulcer margin was taken as standard procedure for histological examinations to exclude malignancy and to detect *Helicobacter pylori (H. pylori)* infection (Giemsa stain) [17], [18]. Mucosal tissue (totally about 15 mg, two pieces of samples) from the ulcer margin and non-ulcer areas (antrum) was taken respectively via endoscopic biopsy and frozen immediately in liquid nitrogen, then stored at −80°C until further analysis. Ulcers were defined as mucosal breaks ≥3 mm [17], [18]. All endoscopy procedures were performed and photocopied by two senior endoscopists (Luo and Hou), and the diagnosis of gastric ulcer was confirmed by another two endoscopists who were blinded to the clinical status of the subjects.

### Isolation of mRNA and Real-time PCR

The total RNA was extracted from gastric mucosa using 500 µl TRIzol reagent (Invitrogen, Carlsbad, USA) according to the manufacturer's protocol. The RNA was further purified using 100 µl chloroform treatment, and then precipitated using 250 µl isopropanol before the complementary DNA synthesis. The genomic DNA contaminated in the extracted RNA was destroyed using DNaseI (Invitrogen, USA) at room temperature for 15 min. The quality of the isolated RNA was verified by electrophoresis on 1.0% agarose-formaldehyde gel, and its quantity was determined by measuring its absorbance at wavelengths of 260- and 280-nm. The single-stranded complementary DNA was synthesized using GoScript™ Reverse Transcriptase (Promega, USA) and oligo dT primer [19].

To quantify the gene expressions of VEGF, VEGFR1, VEGFR2, PDGFA, PDGFB, PDGFRA, PDGFRB, bFGF, FGFR1, FGFR2, quantitative real-time polymerase chain reactions (PCR) were performed using a LightCycler®480 (Roche Applied Science, Indianapolis, IN). The sequences of forward and reverse primers are described in Table 1.

**Table 1:** The sequences of forward and reverse primers for real-time transcriptase- polymerase chain reaction.

Growth factors or receptors		Sequences of primers	product size
VEGF	forward	5′- TCCTCACACCATTGAAACC-3′	79 bp
	reverse	5′- TGGAGGAAGGTCAACCACT-3′	
VEGFR1	forward	5′-AGGCAAGCGCAGGTTCAC-3′	130 bp
	reverse	5′-AAGGCTTCGTGTCAAACTCTAGATG-3′	
VEGFR2	forward	5′-CAAAGGGTGGAGGTGACTGAGT-3′	110 bp
	reverse	5′-GTTTCCCGGTAGAAGCACTTGT-3′	
PDGFA	forward	5′-AGCCCGTTTGTGGCTGAGT-3′	110 bp
	reverse	5′-CACAGACAGAAGCGGCAATG-3′	
PDGFRA	forward	5′-TGCTATCGGCAGATGATGCT-3′	140 bp
	reverse	5′-GGCCAATCTGGCTCAGTCTTC-3′	
PDGFB	forward	5′-CCCAGCAGCTCAAGAAGAAAA-3′	110 bp
	reverse	5′-CAAAGAGCGACCCCATCAGT-3′	
PDGFRB	forward	5′-GCCAGCTACCCCTCAAGGA-3′	130 bp
	reverse	5′-GGATGAGGCAACACTGCTCAA-3′	
bFGF	forward	5′-TCCTGTGTAAACTGCTGGAAGTTCT-3′	150 bp
	reverse	5′-TGTGAGTGGATGGATCTCAATGA-3′	
FGFR1	forward	5′-ACCTGCCTGGGTTTCCCTATAG-3′	110 bp
	reverse	5′-GTCCTACATTCAAAGGCGCTTT-3′	
FGFR2	forward	5′-TTGGTGTGCAACCCTGTCAT-3′	120 bp
	reverse	5′-TCAACTAAGGTCTGTCCTCAAGGA -3′	
GAPDH	forward	5′- GGGTGTGAACCATGAGAAGT -3′	135 bp
	reverse	5′- ACTGTGGTCATGAGTCCTTC-3′	

VEGF: vascular endothelial growth factor, VEGFR1: VEGF receptor 1, VEGFR2: VEGF receptor 2, PDGFA: platelet derived growth factor A, PDGFRA: PDGF receptor A, PDGFRB: PDGF receptor B, bFGF: basic fibroblast growth factor, FGFR1: fibroblast growth factor receptor 1, FGFR2: FGF receptor 2.

GAPDH : glyceraldehydes- 3-phosphate dehydrogenase.

Real-time quantitative PCR was performed on the LightCycler®480 using GoTag® qPCR Master Mix (Promega, USA) according to the manufacturer’s instructions. The quantitative PCR consisted of an initial hold at 95°C for 10 min, then 45 cycles of 95°C for 15 s and 54°C for 1 min, with 40 ng of template cDNA and 2 µM of primer being used for each sample. The signals from each sample were normalized to values obtained for housekeeping gene GAPDH, which was run simultaneously with the experimental samples. A comparative threshold cycle (Ct) method (ΔΔCt) was used to calculate the relative gene expressions (fold change) between test and reference samples [19]. Controls without the RT step or without the template were included for each primer pair to check for any contaminants. As another quality control measure, the melting (dissociation) curves of PCR reactions were monitored to ensure that there was only a single PCR product and no primer dimmers [19]. All the measurement was duplicate in each sample.

### Statistical Analysis

Data were expressed as means±standard deviation (SD) and results were compared between groups using the chi-square test, Fisher’s exact test, Student’s t test, or non-parametric Mann-Whitney U test, when appropriate. Correlations between the two groups were analyzed using Spearman’s correlation method. Non-parametric Wilcoxon signed rank tests were used to compare continuous data for the same patient between ulcer and non-ulcer areas. All statistical analyses were performed using SPSS for Windows version 14.0 (SPSS Inc., Chicago, IL, USA). All *p* values were two-tailed and a value less than 0.05 was considered statistically significant.

## Results

### Demographic Data

From February 2010 to January 2012, 55 eligible cirrhotic and 55 eligible non-cirrhotic patients who had endoscopically proven gastric ulcers were enrolled consecutively. Of the total 110 enrollees, the mean age was 60.3±11.8 years (range, 27–86 years). Sixty-nine (62.7%) patients were male and 41 (37.3%) were female. The demographic data of all patients are shown in Table 2. The two groups were comparable in terms of gender, ulcer size, hemoglobin, serum creatinine and infection rate of *H. pylori.* However, the cirrhotic group were younger in age and had lower platelet count and serum albumin than those of the non-cirrhotic group (p<0.05) (Table 2).

**Table 2:** Demographic data between cirrhotic patients with gastric ulcers and non-cirrhotic patients with gastric ulcers.

	Cirrhotic patients N = 55	Non-cirrhotic patients N = 55	p value
Sex (M/F)	37∶18	32∶23	0.430
Age (years old)	58±13	62±11	0.042
Ulcer size (mm)	7.1±3.1	7.9±3.8	0.337
Hemoglobin (g/dl)	11.3±2.4	10.8±2.3	0.449
Platelet (1000/mm^3^)	95.2±43.3	213.8±81.9	<.001
Creatinine (mg/dl)	1.33±1.83	1.41±1.22	0.333
Albumin (g/dL)	3.1±0.5	3.7±0.7	<.001
Helicobacter pylori+(%)	18 (32.7%)	25 (45.5%)	0.241

Value are means ± S.D.

### mRNA Expressions of Proangiogenic Growth Factors and their Receptors

**Figure 1:**
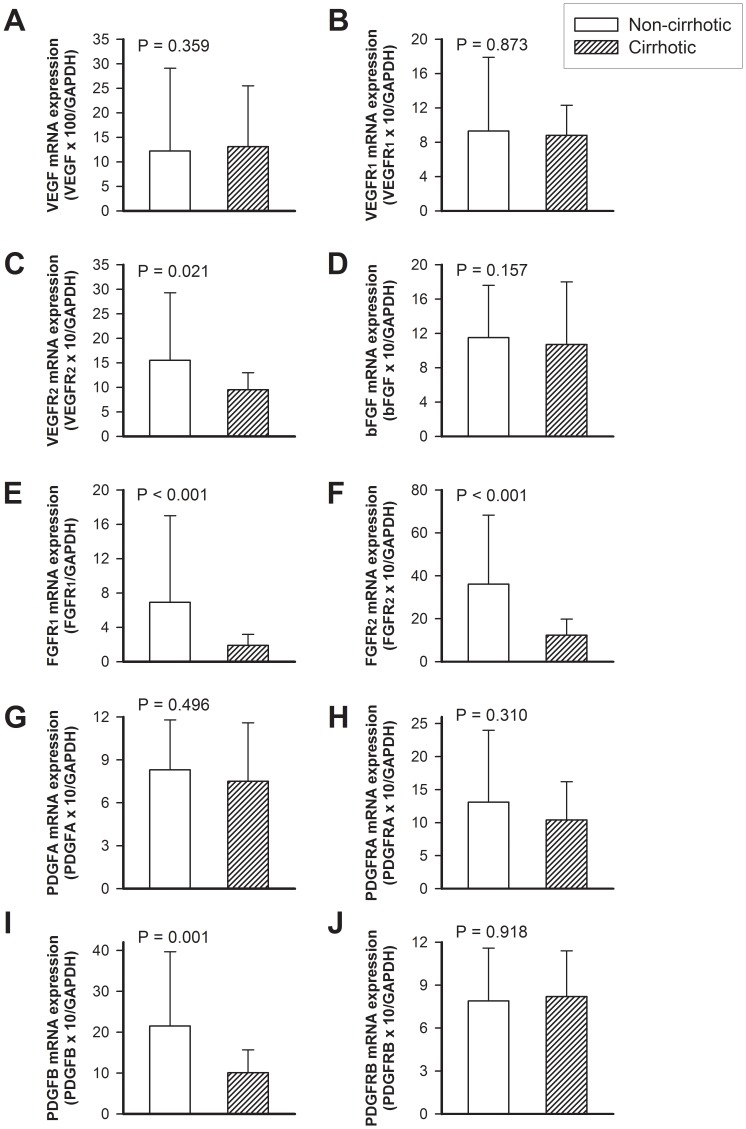
The mRNA expressions of proangiogenic growth factors (VEGF, bFGF, PDGF) and their receptors (VEGFR1, VEGFR2, FGFR1, FGFR2, PDGFRA, PDGFRB) in gastric ulcer margins between the cirrhotic and non-cirrhotic groups.

There were no significant differences in the mRNA expressions of proangiogenic growth factors (VEGF, bFGF, PDGF) and their receptors (VEGFR1, VEGFR2, FGFR1, FGFR2, PDGFRA, PDGFRB) over the non-ulcer areas of gastric mucosa between the cirrhotic and non-cirrhotic groups (Table 3). Over the ulcer margin of gastric mucosa, the cirrhotic group and non-cirrhotic group were comparable in the expressions of VEGF, VEGFR1, bFGF, PDGFA, PDGFRA, and PDGFRB (Fig. 1). However, the cirrhotic group had lower mRNA expressions of PDGFB, VEGFR2, FGFR1, and FGFR2 than the non-cirrhotic group (Fig. 1).

**Table 3:** The mRNA expression of proangiogenic growth factors (VEGF, bFGF, PDGF) and their receptors (VEGFR1, VEGFR2, FGFR1, FGFR2, PDGFRA, PDGFRB) over the non-ulcer part of gastric mucosa.

	Cirrhoticpatients	Non-cirrhotic patients	p value
VEGF×100/GAPDH	12.9±11.3	13.3±15.7	0.495
bFGF×10/GAPDH	7.4±3.9	7.9±4.3	0.330
PDGFA×10/GAPDH	8.4±3.4	8.5±3.2	0.926
PDGFB×10/GAPDH	10.5±5.1	9.9±4.0	0.593
VEGFR1×10/GAPDH	10.3±6.7	9.5±6.8	0.332
VEGFR2×10/GAPDH	9.7±5.4	9.4±5.3	0.819
FGFR1/GAPDH	1.8±1.5	2.2±2.1	0.605
FGFR2×10/GAPDH	14.7±11.0	14.2±9.6	0.271
PDGFRA×10/GAPDH	10.6±5.1	10.3±9.9	0.204
PDGFRB×10/GAPDH	4.8±5.3	3.8±4.2	0.855

VEGF: vascular endothelial growth factor, VEGFR1: VEGF receptor 1, VEGFR2: VEGF receptor 2, PDGFA: platelet derived growth factor A, PDGFRA: PDGF receptor A, PDGFRB: PDGF receptor B, bFGF: basic fibroblast growth factor, FGFR1: fibroblast growth factor receptor 1, FGFR2: FGF receptor 2.

GAPDH : glyceraldehydes- 3-phosphate dehydrogenase Value is means ± S.D.

**Figure 2:**
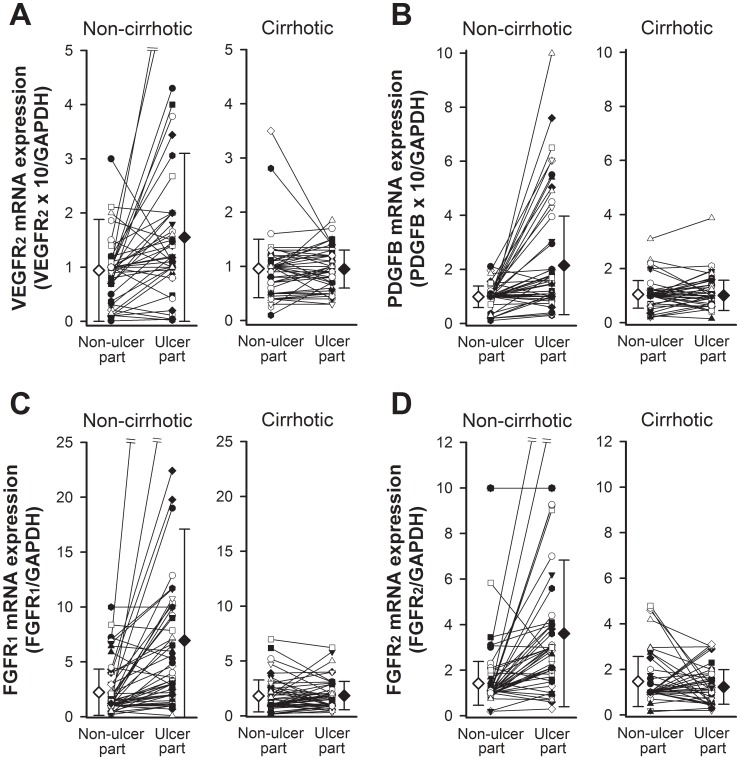
Significant elevation of the mRNA expressions of PDGFB, VEGFR2, FGFR1, and FGFR2 in the ulcer margin was noted compared to the non-ulcer areas (p<0.05) in the non-cirrhotic group, but not in the cirrhotic group.

Further analysis using Wilcoxon signed rank tests was performed to compare data for the same patient between non-ulcer and ulcer margin areas. There were significant elevations in the mRNA expressions of bFGF and PDGFRB in the ulcer margin areas compared to the non-ulcer areas in both the non-cirrhotic and cirrhotic patients (Table 4). There were no significant elevations in the mRNA expressions of VEGF, VEGFR1, PDGFA, and PDGFRA in the ulcer margin areas compared to the non-ulcer areas in both the non-cirrhotic and cirrhotic patients (Table 4). There were significant elevations in the mRNA expressions of VEGFR2, PDGFB, FGFR1, and FGFR2 in the ulcer margin areas compared to the non-ulcer areas (p<0.05) in the non-cirrhotic group, but there were no significant elevations of the mRNA expressions of PDGFB, VEGFR2, FGFR1, and FGFR2 in the ulcer margin areas compared to the non-ulcer areas in the cirrhotic group (Fig. 2 and Table 4).

**Table 4:** Compare the expression of proangiogenic growth factors (VEGF, bFGF, PDGF) and their receptors (VEGFR1, VEGFR2, FGFR1, FGFR2, PDGFRA, PDGFRB) over the non-ulcer part and ulcer margin of gastric mucosa.

Non-cirrhotic group	Non-ulcermucosa	Ulcer marginmucosa	p value
VEGF×100/GAPDH	13.3±15.7	12.3±16.9	0.850
bFGF×10/GAPDH	7.9±4.3	11.5±6.1	0.034
PDGFA×10/GAPDH	8.5±3.2	8.3±3.5	0.538
PDGFB×10/GAPDH	9.9±4.0	21.5±18.2	<.001
VEGFR1×10/GAPDH	9.5±6.8	9.3±8.6	0.776
VEGFR2×10/GAPDH	9.4±5.3	15.5±13.8	<.001
FGFR1/GAPDH	2.2±2.1	6.9±9.7	<.001
FGFR2×10/GAPDH	14.2±9.6	36.1±32.1	<.001
PDGFRA×10/GAPDH	10.3±9.9	12.1±10.9	0.063
PDGFRB×10/GAPDH	3.8±4.2	7.9±3.8	<.001
**Cirrhotic group**	**non-ulcer mucosa**	**ulcer margin mucosa**	**p value**
VEGF×100/GAPDH	12.9±11.3	13.1±12.4	0.959
bFGF×10/GAPDH	7.4±3.9	10.7±7.3	0.003
PDGFA×10/GAPDH	8.4±3.4	7.5±4.1	0.148
PDGFB×10/GAPDH	10.5±5.1	8.2±3.2	0.358
VEGFR1×10/GAPDH	10.3±6.7	8.8±3.5	0.191
VEGFR2×10/GAPDH	9.7±5.4	9.5±3.5	0.654
FGFR1/GAPDH	1.8±1.5	1.9±1.3	0.476
FGFR2×10/GAPDH	14.7±11.0	12.4±7.6	0.510
PDGFRA×10/GAPDH	10.6±5.1	10.4±5.8	0.969
PDGFRB×10/GAPDH	4.8±5.3	8.2±3.2	0.001

VEGF: vascular endothelial growth factor, VEGFR1: VEGF receptor 1, VEGFR2: VEGF receptor 2, PDGFA: platelet derived growth factor A, PDGFRA: PDGF receptor A, PDGFRB: PDGF receptor B, bFGF: basic fibroblast growth factor, FGFR1: fibroblast growth factor receptor 1, FGFR2: FGF receptor 2.

GAPDH : glyceraldehydes- 3-phosphate dehydrogenase Value is means ± S.D.

### Correlation between Proangiogenic Growth Factors/receptors and Platelet Count

**Figure 3:**
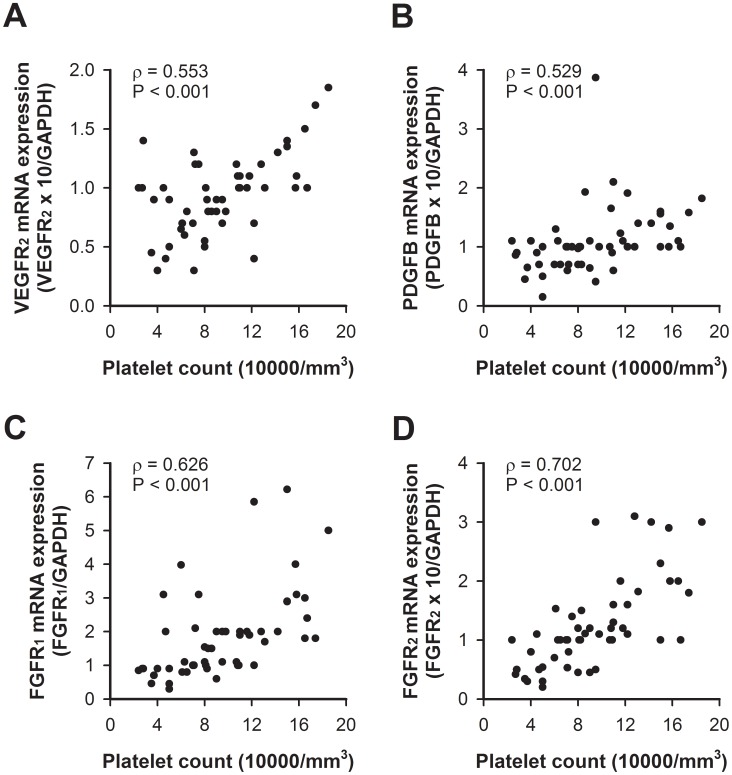
The correlation between the mRNA expressions of PDGFB, VEGFR2, FGFR1, and FGFR2 in the gastric ulcer margins and the platelet count in the cirrhotic patients (p>0.5, p<0.001).

The mRNA expressions of PDGFB, VEGFR2, FGFR1, and FGFR2 in the ulcer margin areas of the cirrhotic patients had good correlation with the platelet count (ρ>0.5, p<0.001, Fig. 3).

## Discussion

We found that cirrhotic patients had diminished mRNA expressions of proangiogenic growth factors (PDGFB) and their receptors (VEGFR2, FGFR1, and FGFR2) in gastric ulcer margins compared with those of the non-cirrhotic patients. These diminished expressions of PDGFB, VEGFR2, FGFR1, and FGFR2 were well correlated with the degree of thrombocytopenia in the cirrhotic patients. These findings imply impaired gastric ulcer healing in cirrhotic patients and provide a possible explanation for the higher occurrence of peptic ulcers in cirrhotic patients.

Factors that can influence gastric ulcer healing include age, drinking, smoking, use of NSAIDs, aspirin, steroids, clopidogrel, ticlopidine, proton pump inhibitors, histamine receptor 2 antagonists, misoprostol, status of *H. pylori* infection, and nutritional condition [20]. We excluded patients who drank alcohol, smoked, took the aforementioned medications, and those with unstable disease activity, poor general condition, or underlying malignancies during enrollment to avoid these confounding factors. The cirrhotic patients were younger in age, and had lower serum albumin compared to the non-cirrhotic patients. However, they were comparable in serum creatinine, gastric ulcer size, and the rate of *H. pylori* infection.

PDGF, a glycoprotein, is stored in platelets and is released upon stimulation. PDGF accounts for approximately 50% of platelet-derived mitogenic activity [21], stimulating the proliferation of arterial smooth cells and fibroblasts. PDGF, together with bFGF, plays a major role in the reconstruction of connective tissue including angiogenesis and accelerates ulcer healing [21], [22]. The good correlation between platelet count and PDGFB expression suggests that thrombocytopenia in cirrhotic patients may have an impact on PDGFB secretion thereby influencing gastric ulcer healing.

bFGF, by activating FGFR-ERK signal transduction pathway, has been shown to significantly accelerate ulcer healing via increasing the density of microvessels in the ulcerated tissue and promoting epithelial cell proliferation [19], [22], [23]. Our findings showed that mRNA expressions of bFGF were significantly increased in gastric ulcer margin of both cirrhotic and non-cirrhotic patients (Table 4). However, mRNA expressions of FGFR1 and FGFR2 were significantly decreased in the gastric ulcer margins of the cirrhotic patients compared to those of the non-cirrhotic patients, and that the mRNA expression of FGFR was well correlated with the platelet count in cirrhotic patients. Due to the limited amount of gastric samples, we could not clarify the relationship between the bFGF-FGFR-ERK signal transduction pathway, angiogenesis and epithelial cell proliferation in the cirrhotic patients. Further studies using a cirrhotic rat model are needed to clarify the role of thrombocytopenia in bFGF-FGFR-ERK signaling, angiogenesis, and ulcer healing.

The presence of VEGF mRNA and protein in megakaryocytes provides strong evidence that VEGF synthesis during thrombopoiesis is the origin of platelet VEGF [24]. VEGF, which is stored in platelet α-granules, is secreted from platelet aggregation induced by thrombin, collagen or adenosine diphosphate [25]. VEGF and its receptor, VEGFR, significantly accelerates gastric ulcer healing by enhancing angiogenesis including endothelial cell proliferation, migration, and tube formations at the ulcer site [22], [26]. One animal study showed that platelets accelerated gastric ulcer healing through the presentation of VEGF [27]. However, our study only showed that the mRNA expression of VEGFR2 increased significantly in the ulcer margins of the non-cirrhotic patients but not in the cirrhotic patients who had significantly lower platelet counts. Neither VEGF nor PDGFA was elevated in gastric ulcer margin of the cirrhotics and non-cirrhotics in our study, which was not consistent with previous studies showing increased VEGF and PDGF expression during gastric ulcer healing both in humans and in experimental animals [11], [12], [22], [26]. The possible explanation is that we just took superficial (non-deep) biopsy with small forceps to get mucosa tissue of ulcer margin both in cirrhotics and non-cirrhotics to avoid post-biopsy bleeding in cirrhotics under ethical consideration.

This is the first clinical study to show diminished mRNA expressions of proangiogenic growth factors (PDGFB) and their receptors (VEGFR2, FGFR1, and FGFR2) in gastric ulcer margins of cirrhotic patients, and that these diminished expressions were well correlated with lower platelet count in these patients. Platelets are important in ulcer healing [12]. Thrombocytopenic rats have been shown to exhibit delayed gastric ulcer healing when compared to healthy rats [28]. This retarded ulcer healing could be reversed by platelet transfusions from healthy rats, and increasing the number of circulating platelets in the rats improved gastric ulcer healing through administration of thrombopoeitin [27], [28].

Unexpectedly, diminished expression of VEGF and PDGFA-proangiogenic growth factors was not found, whereas diminished expression of VEGFR2, FGFR1 and FGFR2 -proangiogenic growth factors receptors was noted. All the cirrhotics we enrolled in this study had history of esophageal variceal bleeding and had received variceal ligation and regular follow-up, which implies they have more severe portal hypertension. It is likely that patients with a lower platelet count had more severe portal hypertension and that the vascular derangements secondary to portal hypertension lead to the decreased expression of the receptors for proangiogenic factors like FGFR1, FGFR2, and VEGFR2. Therefore, further study using experimental animals is needed to clarify the issue.

There are several limitations to the present study. First, although the average ulcer size was comparable in both groups, sampling bias (biopsies from gastric ulcer margins) could not be avoided. Second, there were a limited number of gastric samples from each subject to clarify the relationships between the expressions of proangiogenic growth factors, their receptors, signal transduction pathways for angiogenesis and cell proliferation using Western blotting or other methods. Third, ulcer healing and the expression of growth factors are dynamic. It is therefore impossible and unethical to imitate animal studies [28] and evaluate changes in proangiogenic growth factors, their receptors, and mechanisms of the healing process after transfusion of platelets in cirrhotic patients or after depletion of platelets in non-cirrhotic patients.

### Conclusion

The pathogenesis of PUD in cirrhotic patients is complex, and mechanisms involving delayed ulcer healing may be a major reason. We found that diminished mRNA expressions of proangiogenic growth factors (PDGFB) and their receptors (VEGFR2, FGFR1, and FGFR2) in gastric ulcer margins were well correlated with the degree of thrombocytopenia in the cirrhotic patients. These findings suggested that diminished activity of proangiogenic factors and their receptors may contribute to the pathogenesis of gastric ulcers in cirrhotic patients.
